# The requirement for mitochondrial respiration in cancer varies with disease stage

**DOI:** 10.1371/journal.pbio.3001800

**Published:** 2022-09-23

**Authors:** Colin Sheehan, Alexander Muir

**Affiliations:** Ben May Department for Cancer Research, University of Chicago, Chicago, Illinois, United States of America

## Abstract

The roles for glycolytic and respiratory metabolism in supporting in vivo tumor growth in different contexts are not well understood. In this issue of *PLOS Biology*, a new study reveals that primary and metastatic tumors demonstrate divergent metabolic requirements.

A long-standing question in tumor biology is what role mitochondrial metabolism plays in fueling tumor biology. To address this question, Bennett and colleagues perform an in vivo CRISPR-interference screen in a mouse lung cancer model to assess the functional consequences of different metabolic perturbations as tumors grow and progress to metastatic lesions [1]. In doing so, the authors surprisingly find that inhibiting mitochondrial metabolism has different effects depending on the stage of tumor progression. These findings argue that mitochondrial respiration plays distinct roles at different stages of tumor progression, suggesting that there is not a single cancer metabolic phenotype, but rather a spectrum of metabolic phenotypes that change over the course of disease progression.

Cells generate energy in the form of ATP using 2 major metabolic routes—glycolysis and oxidative phosphorylation (OXPHOS). OXPHOS, also called respiration, is a metabolic process wherein electrons generated by the breakdown of nutrients are transferred onto oxygen by a collection of protein complexes in the mitochondria, known as the electron transport chain. In the process, the free energy generated from reducing oxygen powers the synthesis of ATP via an electrochemical proton gradient.

In seminal studies of how cancers use nutrients to generate energy, cancer cells were found to often have intrinsically reduced cellular respiration even in conditions with abundant oxygen [2,3]. Given these observations, Otto Warburg famously speculated that a critical feature of cancer cells—and even the origin of the disease—is dysfunctional or “broken” mitochondria, resulting in a subsequent reliance on glycolysis for ATP regeneration [3]. However, recent work suggests that despite decreased respiration in cancer cells, this metabolic pathway still plays an essential role in supporting tumorigenesis. For example, multiple studies have now demonstrated that respiration-deficient cancer cells have severely compromised ability to form tumors [4,5]. Strikingly, respiration-incompetent cancer cells lacking mitochondrial DNA fail to establish tumors until they capture functional mitochondria from surrounding host stromal cells [6]. Given the apparent essentiality of respiration for tumor growth, there are multiple ongoing efforts to use respiration inhibitors in the treatment of various cancers [7].

Thus, our view of the role of respiration in cancer biology has shifted from this metabolic pathway being inactive in cancer to being essential for tumor growth. However, our knowledge of respiration in cancer biology is mostly limited to one aspect of tumor progression, growth of the primary tumor. Far less is known about what role respiration plays during disease progression to metastasis. To answer this question, the authors performed an in vivo CRISPR-interference screen using a custom sgRNA library targeting glycolytic and respiratory genes to determine the relative essentiality of these metabolic pathways across disease progression, assessing both the primary tumor and distant metastases sites. Consistent with previous studies, there is strong selection against guide RNAs targeting genes involved in mitochondrial protein translation and cellular respiration in primary tumors. More surprisingly, the authors report that there was far less selection against respiration-targeting guide RNAs at metastatic lesions, a strong indication of differential respiratory requirements between primary tumor growth and metastatic dissemination (Fig 1). Confirming this interpretation of the screening results, individual knockdown of these genes favored metastatic tumor formation over primary tumor growth. Thus, these findings suggest that lung cancer metastases, in contrast to primary tumors, may be promoted by decreased mitochondrial respiration and indeed a recent study has found aberrant mitochondrial structure and function in metastatic lung tumors [8]. Altogether, this work demonstrates that there are unique metabolic requirements for different stages of disease progression and highlights the importance of assessing metabolic function and requirement at different points in time within the same tumor model [9].

**Fig 1:**
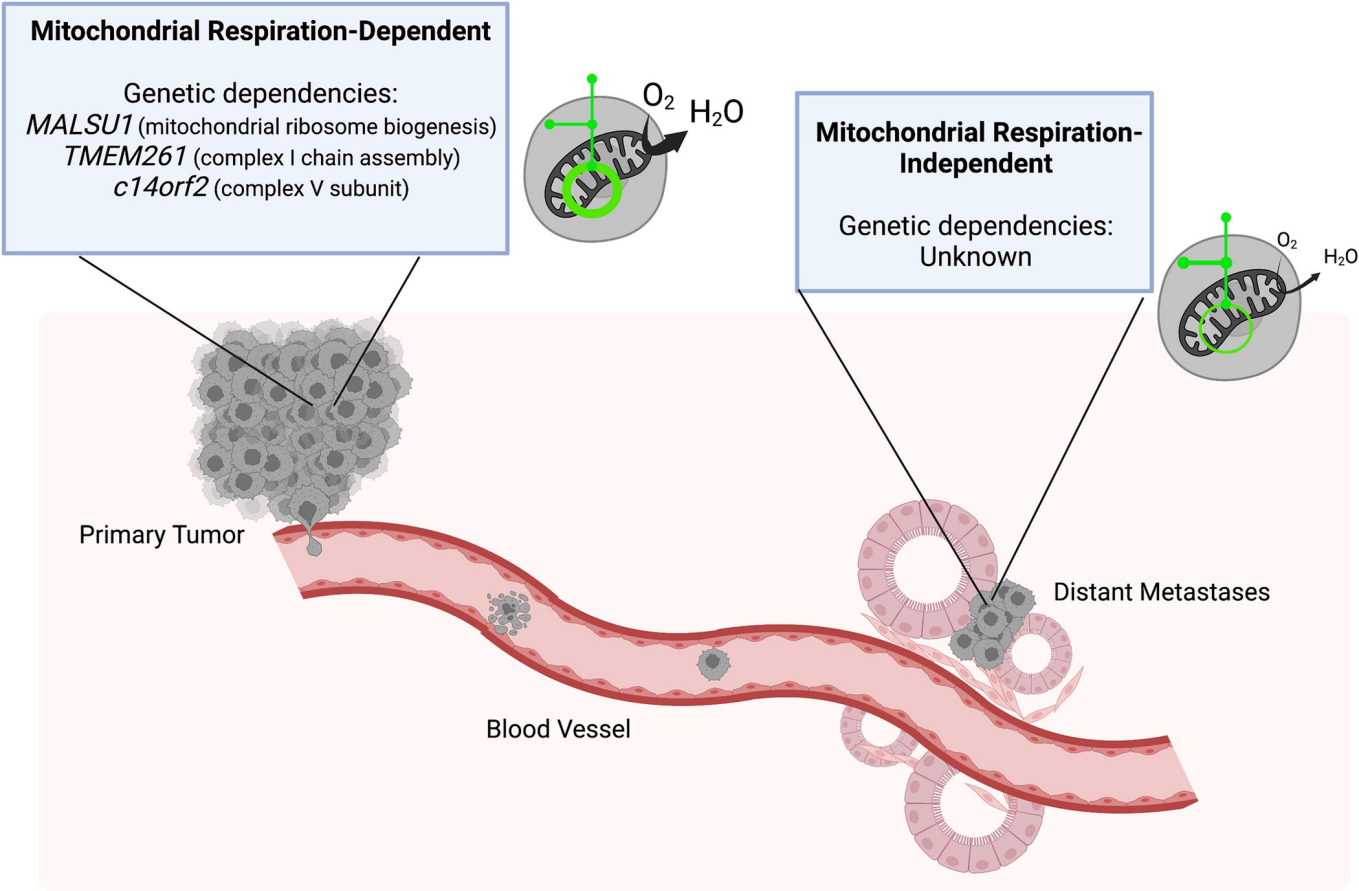
Primary tumor growth and metastasis possess unique metabolic dependencies. Cancer cells growing at the primary tumor site are dependent on genes involved in mitochondrial protein translation and respiration (*MALSU1*, *TMEM261*, *c14orf2*) (left). In contrast, there is surprisingly low dependence on these genes for the formation of distant metastases (right). Figure created using BioRender.

The findings of this study indicate that our understanding of the role of respiration in cancer biology is far from complete and open many questions. First, why is respiration dispensable or even inhibitory for lung cancer metastasis, yet so critical for primary tumor growth? While best known for its role in maintaining cellular ATP via OXPHOS, respiration also performs other critical functions including enabling multiple biosynthetic pathways, balancing cellular redox, and producing signaling molecules [10]. Indeed, others have suggested the critical function of respiration for primary tumor growth is in supporting these latter functions, rather than the production of ATP by OXPHOS [5]. To distinguish between these possible explanations for the in vivo requirement of respiratory genes in lung tumors, the authors characterized the metabolic phenotypes of lung cancer cells subjected to CRISPR knockdown of respiratory genes. While knockdown of OXPHOS components produced varying changes to the redox potential, there was a shared decrease in cellular respiration and ATP levels among all the respiratory genes tested. This suggests that ATP regeneration by OXPHOS may be important in primary tumor growth, but less so for metastatic lesions. However, more studies probing metabolic constraints imposed on primary and metastatic tumors in vivo by OXPHOS inhibition will be required to understand the precise role respiration is playing in lung cancer metabolism during these different states of disease progression.

A second interesting question is if mitochondrial metabolism is required for different tumor types that migrate to different metastatic destinations. Recent studies in other metastatic tumor types, including breast, pancreatic, and oral carcinomas suggest cancer cells up-regulate and require respiration for metastatic progression [11–13]. This suggests the metabolic requirements for metastasis may be context dependent. Both cancer cell-intrinsic differences (e.g., genetic lesions and tissue-of-origin) and cell-extrinsic environmental differences at different metastatic sites (e.g., local nutrient availability) could influence the metabolic requirements for metastasis. In addition, different stages of metastasis (i.e., cell invasion, dissemination, micrometastatic colonization, macrometastatic outgrowth) could have different requirements for mitochondrial respiration. Thus, systematic screening approaches—such as those used by the authors—in cancer models that recapitulate all these stages of cancer progression will be needed to fully understand the role that mitochondrial metabolism plays over the course of disease progression. Indeed, such studies will be critical to determine how therapies targeting mitochondrial respiration might impact different tumors at different disease stages.
